# The prevalence and phenotypic range associated with biallelic *PKDCC* variants

**DOI:** 10.1111/cge.14324

**Published:** 2023-03-10

**Authors:** Alistair T. Pagnamenta, Rebecca S. Belles, Bonnie Anne Salbert, Ingrid M. Wentzensen, Maria J. Guillen Sacoto, Francis Jeshira Reynoso Santos, Alesky Caffo, Matteo Ferla, Benito Banos‐Pinero, Karolina Pawliczak, Mina Makvand, Hossein Najmabadi, Genomics England Research Consortium, Reza Maroofian, Tracy Lester, Ana Lucia Yanez‐Felix, Camilo E. Villarroel‐Cortes, Fan Xia, Khowla Al Fayez, Amal Al Hashem, Deborah Shears, Melita Irving, Amaka C. Offiah, Ariana Kariminejad, Jenny C. Taylor

**Affiliations:** ^1^ NIHR Biomedical Research Centre, Wellcome Centre for Human Genetics University of Oxford Oxford UK; ^2^ Geisinger Health System Danville Pennsylvania USA; ^3^ GeneDx Gaithersburg Maryland USA; ^4^ Joe DiMaggio Children's Hospital Hollywood Florida USA; ^5^ Division of Human Genetics Children's Hospital of Philadelphia Philadelphia Pennsylvania USA; ^6^ Oxford Genetics Laboratories Oxford University Hospitals NHS Foundation Trust, The Churchill Hospital Oxford UK; ^7^ South East Genomic Laboratory Hub Guy's Hospital London UK; ^8^ Kariminejad‐Najmabadi Pathology & Genetics Center Tehran Iran; ^9^ Genetics Research Center University of Social Welfare & Rehabilitation Science Tehran Iran; ^10^ Department of Neuromuscular Diseases UCL Queen Square Institute of Neurology, University College London London UK; ^11^ Human Genetics Department National Pediatrics Institute Mexico City Mexico; ^12^ Baylor Genetics Houston Texas USA; ^13^ Department of Pediatrics, Division of Clinical Genetic and Metabolic Medicine Prince Sultan Medical Military City Riyadh Saudi Arabia; ^14^ Oxford Centre for Genomic Medicine Oxford University Hospitals NHS Foundation Trust Oxford UK; ^15^ Department of Clinical Genetics Guy's and St Thomas' NHS Foundation Trust London UK; ^16^ Department of Oncology & Metabolism University of Sheffield Sheffield UK

**Keywords:** bone diseases, medical genetics, skeletal dysplasia, whole genome sequencing

## Abstract

*PKDCC* encodes a component of Hedgehog signalling required for normal chondrogenesis and skeletal development. Although biallelic *PKDCC* variants have been implicated in rhizomelic shortening of limbs with variable dysmorphic features, this association was based on just two patients. In this study, data from the 100 000 Genomes Project was used in conjunction with exome sequencing and panel‐testing results accessed via international collaboration to assemble a cohort of eight individuals from seven independent families with biallelic *PKDCC* variants. The allelic series included six frameshifts, a previously described splice‐donor site variant and a likely pathogenic missense variant observed in two families that was supported by in silico structural modelling. Database queries suggested that the prevalence of this condition is between 1 of 127 and 1 of 721 in clinical cohorts with skeletal dysplasia of unknown aetiology. Clinical assessments, combined with data from previously published cases, indicate a predominantly upper limb involvement. Micrognathia, hypertelorism and hearing loss appear to be commonly co‐occurring features. In conclusion, this study strengthens the link between biallelic inactivation of *PKDCC* and rhizomelic limb‐shortening and will enable clinical testing laboratories to better interpret variants in this gene.

## Graphical abstract

Although *PKDCC* variants have been implicated in rhizomelic shortening of limbs, the original association was based on just two patients. In this study, using data from the 100 000 Genomes Project and international collaboration, we describe a new cohort of eight individuals from seven independent families with biallelic *PKDCC* variants.

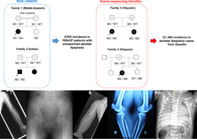

## 1 INTRODUCTION

The Hedgehog signalling pathway plays a crucial role in normal skeletal development and repair.^1^ Mouse studies suggest that Hedgehog signalling can be regulated by protein kinase domain containing, cytoplasmic (Pkdcc), a secreted protein tyrosine kinase also known as Vertebrate lonesome kinase (Vlk). This protein phosphorylates several targets such as extracellular matrix metalloproteases and the Smoothened transmembrane protein, an inhibitor of Notched, the Hedgehog receptor. This regulation of Hedgehog signalling is crucial during skeletal development.^2^, ^3^ Pkdcc null mice typically die shortly after birth with severe craniofacial/limb skeletal defects.^4^ In 2019, biallelic variants in the human orthologue (*PKDCC*) were identified in patients with skeletal dysplasia.^5^ Although just two unrelated individuals were reported, the condition is listed in OMIM as ‘Rhizomelic limb shortening with dysmorphic features’ (MIM#618821).

Where novel disease‐gene associations are proposed based on so few patients it is important for additional families to be described, even where there is support from pathway analysis and model organisms. Consequently, *PKDCC* was initially classed as ‘Amber’ in the skeletal dysplasia panel on PanelApp. Variants in *PKDCC* were therefore not prioritised by the 100 000 Genomes Project^6^ (100 kGP) and other studies that utilised PanelApp as part of their pipeline.

In this study, genome, exome and panel‐NGS sequencing was performed in tandem with international collaborative efforts to assemble a cohort of seven new families with rare biallelic variants in *PKDCC*. As four of the families were recruited via large clinical databases, this allowed us to estimate the prevalence of this rare condition in suitable patient cohorts. These results strengthen the reported gene‐disease association and in combination with the cases published previously, enable a more precise delineation of the phenotypic spectrum associated with this rare condition.

## 2 METHODS

Families 1 and 2 were sequenced as parent–child trios as part of the 100 kGP. Ethics approval was from Cambridge South REC (14/EE/1112) and the study conforms to recognised standards. Library preparation was using the TruSeq PCR‐free high throughput kit. Genome sequencing data was generated in a single lane of a HiSeqX (Illumina) and 150 bp paired‐end reads were mapped using the iSAAC aligner to GRCh38. Severe consequence homozygous variants were extracted using the ‘tiering_data’ table available within Genomic England's research environment and a 1% population allele frequency threshold was employed. Families 3 and 4 were identified via GeneMatcher (https://genematcher.org). Exome sequencing was performed using the IDT xGen Exome Research Panel v1.0 by GeneDx, as described.^7^ In Family 5, customised exome sequencing was performed using the Illumina NovaSeq platform (Data S1). For Family 6, sequencing had been performed at up to 50x using NGS‐panels comprising 438 genes. The individual from Family 7 underwent clinical exome analysis, as described.^8^ Structural modelling is described in Data S1.

## 3 RESULTS

Using the tiering data generated as part of the 100kGP, we identified a homozygous *PKDCC* variant NM_138370.3:c.939dupA, p.(Leu314Thrfs*29) in a female proband (Family 1) with rhizomelic shortening of the upper limbs and facial dysmorphism. The variant lay within a 16.5 Mb region of homozygosity (ROH), consistent with the documented consanguinity. Both unaffected parents were confirmed to be heterozygous carriers.

Initially, no further biallelic *PKDCC* cases from the 100kGP could be identified. However, following discussion at a multidisciplinary team meeting to review unsolved musculoskeletal cases, *PKDCC* emerged as a strong candidate in Family 2. The proband (F2‐II‐1) presented isolated rhizomelia of upper limbs. Non‐skeletal features included mild neutropenia, hypoplastic pituitary gland on imaging, growth hormone deficiency and low TSH. Teeth showed evidence of enamel pitting and there was mild hypertelorism and a slight metopic ridge. In utero radiological assessment at 20 weeks also indicated that the proband was likely to have a similarly affected sibling and rhizomelic limb‐shortening has now been confirmed postnatally. This last observation made an autosomal‐recessive mode of inheritance more likely. *PKDCC* was one of 70 genes present on the current PanelApp skeletal dysplasia gene list (https://panelapp.genomicsengland.co.uk/panels/309), but absent from the version used at the time of the initial analysis (Table S1). Finally, *PKDCC* lay in a 791 kb ROH (Table S2, discussion in Data S1). These findings prompted manual review of read‐alignments using IGV. A homozygous c.228dupG, p.(Pro77Alafs*95) variant was uncovered, within a 174 bp guanine‐rich region of low complexity.

In Family 3, a homozygous c.606dupG, p.(Leu203Alafs*96) *PKDCC* variant was shared by two sisters with short stature, rhizomelia and bilateral clinodactyly. Both unaffected parents were confirmed to be heterozygous carriers. In Family 4, an individual initially suspected to have pseudoachondroplasia had inherited a c.290_320del31, p.(Leu97Profs*123) variant in *PKDCC* from her unaffected mother alongside a second variant c.492delG, p.(Leu165Serfs*65). The c.492delG was later shown to be inherited from her father using Sanger sequencing, confirming compound‐heterozygosity.

The affected individual in Family 5 (F5‐II‐10) was referred for clinical assessment because of an unknown skeletal dysplasia. His condition included short stature with rhizomelic limb‐shortening, macrocephaly, mild facial dysmorphism, hearing impairment, hoarse voice, genu varum, patellofemoral dislocation and short thumbs (Figure S1). Exome sequencing uncovered a homozygous c.785 T > G, p.(Leu262Arg) variant. Although biparental inheritance was unconfirmed, the parents of F5‐II‐10 are first cousins. The proband's wife (F5‐II‐11; a second cousin), their daughter (F5‐III‐1) and their son (F5‐III‐2) were all heterozygous carriers and the recurrence risk in further pregnancies was therefore 50%.

In Family 6, a targeted approach identified two heterozygous *PKDCC* variants in a male child (F6‐II‐2) with short stature, macrocephaly, rhizomelia and brachydactyly. The first variant c.639 + 1G > T was described previously,^5^ whilst c.785 T > G, p.(Leu262Arg) was the same variant identified in Family 5. Phasing was confirmed to be *in trans* by subsequent testing of parental DNA.

Lastly, exome sequencing identified a homozygous c.380dup, p.(Arg129Alafs*43) variant in individual F7‐II‐5. This girl had an atrial septal defect, micrognathia, hypertelorism, cupped ears with rotated, fleshy ear lobes, an elfin‐like face and rhizomelia. Inferior vermis hypoplasia was also noted but asymptomatic. Family history is significant for a similarly affected older brother.

**FIGURE 1:**
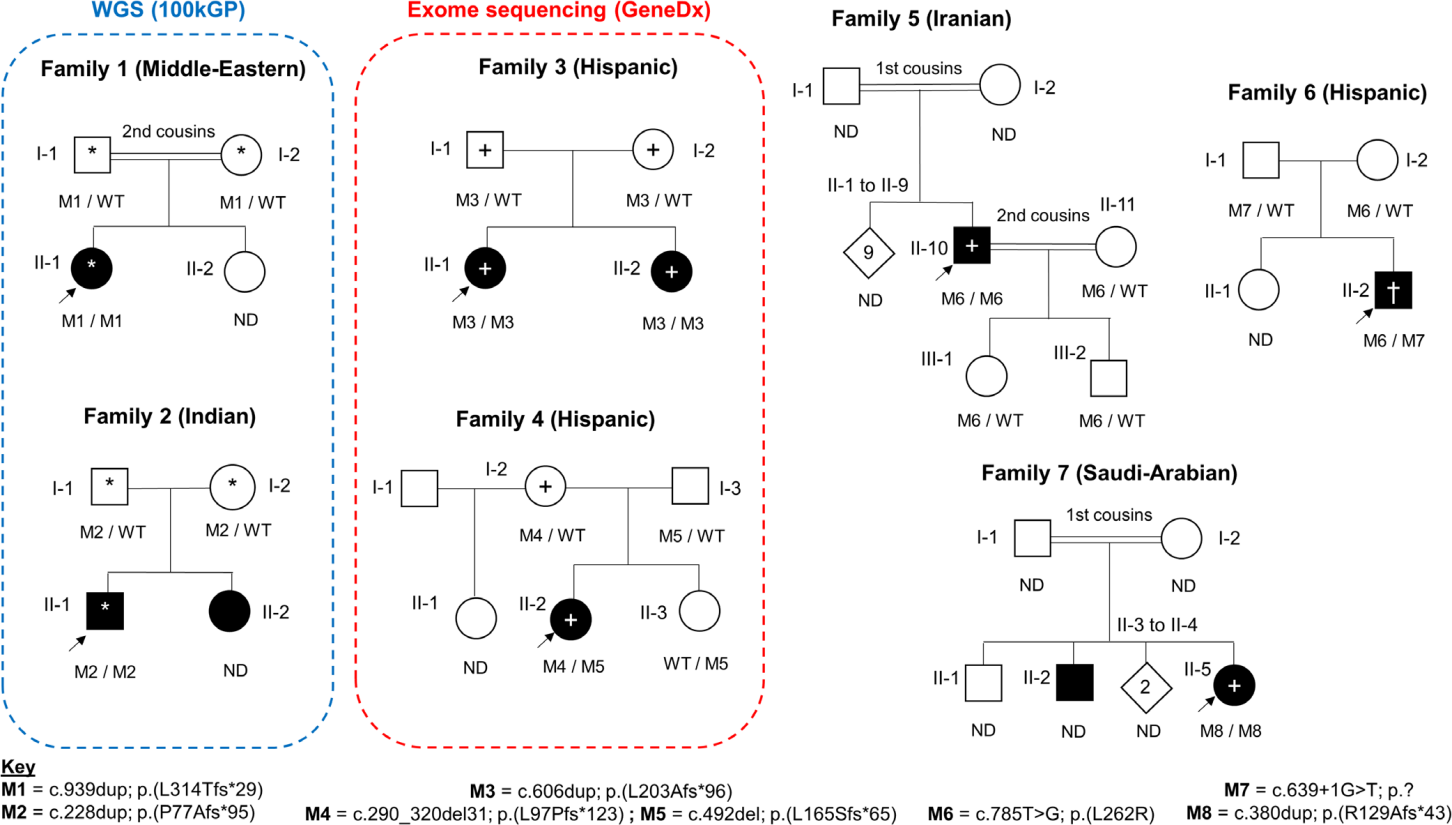
Genetic segregation and pedigree information for eight affected individuals harbouring rare biallelic *PKDCC* variants. Shading indicates upper limb rhizomelia with mild facial dysmorphisms; ND, not determined; WT, wild‐type; *, genome sequencing performed as part of the 100 K Genomes Project, +, exome sequencing, †, gene‐panel testing. Variant coordinates based on NM_138370.3. [Colour figure can be viewed at wileyonlinelibrary.com]

In all families, variant segregation was consistent with an autosomal‐recessive mode of inheritance (Figure 1). Of the eight variants observed across these seven families, all have been validated (Figure S2) and have allele frequencies in gnomAD v2.1.1 of 0–6/158918 (Table S3). Notably, 6/8 variants are frameshifts lying between codons 77‐314. Wild‐type *PKDCC* encodes a protein of 493 amino‐acids and so this set of variants likely result in loss of protein stability/function. Pathogenicity of p.(Leu262Arg) is supported by in silico scores (CADD = 29.1/PolyPhen = ‘probably_damaging’). Residue 262 is a buried leucine 14 Å away from the ATP and magnesium cofactors (Figure S3) and molecular thermodynamic predictions suggest a 2.0 kcal/mol increase (destabilisation) in Gibbs free‐energy of folding. An interactive model is available at https://michelanglo.sgc.ox.ac.uk/r/pkdcc.

## 4 DISCUSSION

**FIGURE 2:**
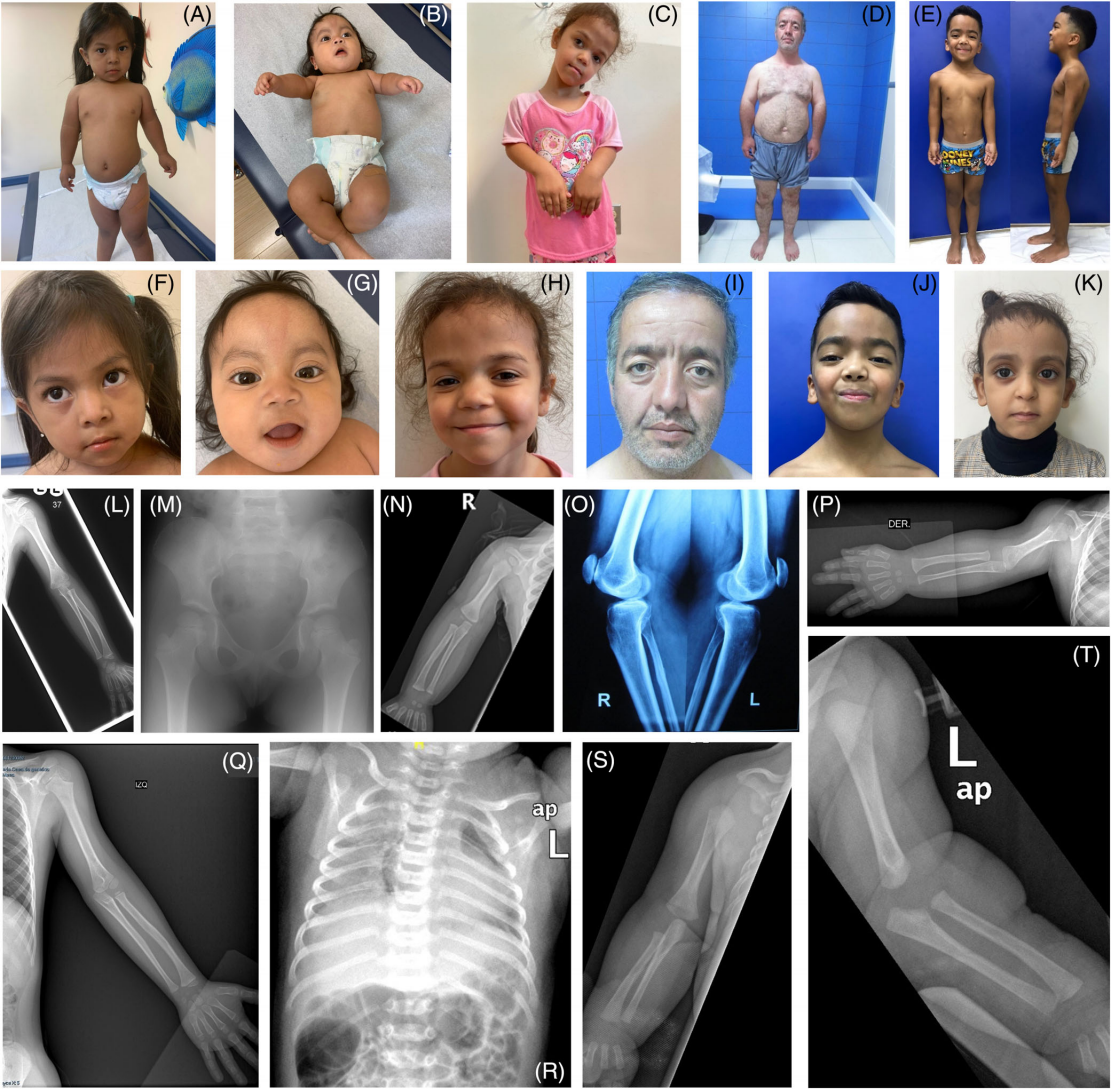
Clinical and radiographic images for individuals with biallelic *PKDCC* variants. Full length (A–E) and facial photos (F–K) of affected individuals from Families 3–7. (A, F) Photos showing F3‐II‐1 (aged 3 years) with rhizomelia of the upper limbs and widely spaced nipples. (B, G) Photos showing F3‐II‐2 (aged 4 months) with thick, arched, eyebrows, nevus flammeus, thin upper lip and a long philtrum. (C, H) Photos showing F4‐II‐2 (aged 5 years) with rhizomelia of the upper limbs, midface hypoplasia, a high forehead, hypertelorism, a thin upper lip and a long philtrum. (D, I) Photos showing F5‐II‐10 with high and broad forehead, flat face, rhizomelia of upper and lower limbs. (E, J) Photos showing F6‐II‐2 (aged 7 years 4 months) with prominent forehead, flat face, low set ears, sloping shoulders, pectus excavatum and rhizomelia. (K) Photo of F7‐II‐5 showing prominent eyes/forehead. (L) Antero‐posterior radiograph of left upper limb in F1‐II‐1 (aged 4 years 10 months). There is ‘mild shortening of the humerus’ (rhizomelia). (M) Antero‐posterior radiograph of pelvis in F1‐II‐1 (aged 4 years 10 months) showing ‘mild coxa valga’. (N) Antero‐posterior radiograph of the right upper limb of F2‐II‐1 (aged 9 months) demonstrating isolated ‘shortening of the humerus’ (rhizomelia). (O) Lateral knee radiographs of individual F5‐II‐10 show superior subluxation of the patellae and bilateral degenerative change with narrowing of the patellofemoral joints (worse on the right). (P, Q) Antero‐posterior radiograph of the upper limbs of F6‐II‐2 (aged 1 year and at 6 years 5 months) demonstrating ‘shortening of the humerus’ (rhizomelia). (R) AP chest and upper limb radiographs taken of F7‐II‐5 at birth show ‘11 ribs’ bilaterally and (S, T) ‘shortening of the humeri’ (rhizomelia). Radiographs annotated using terms from the dREAMS ontology (https://d‐reams.org; inverted commas). [Colour figure can be viewed at wileyonlinelibrary.com]

In this study, international collaborative efforts identified 8 individuals from 7 families with biallelic variants in *PKDCC*. Detailed phenotypic information is provided in Tables 1 and S3. These eight individuals have consistent rhizomelic shortening, predominantly of the upper limbs (Figure 2A–E).

**TABLE 1:** Summary of clinical findings and variants across the 10 individuals with *PKDCC* variants (8 described here and 2 by Sajan et al*.* ^5^).

Clinical features	
Gender	5 female, 3 male, 2 not reported
Age range at last assessment	16 months–43 years
Consanguinity	3/10
Rhizomelia	10/10 (predominantly upper limb)
Short fifth digit	5/9
Micrognathia	5/8
Hearing loss	4/8
Hypertelorism	6/9
Sloping shoulders	3/8
Flat face	5/8 (evident from photos)
High and broad forehead	4/9
Patellofemoral dislocation	3/9
Short stature	8/10
Prominent eyes	3/10
Macrocephaly	3/7

Variant details	
Variant consequences	6 frameshifts, 1 nonsense, 1 splice‐donor (2 families), 1 missense (2 families)
Variant allele‐counts (gnomAD 2.1.1)	0–4
CADD	29.1–41
Genotypes	8 homozygotes, 2 compound‐heterozygotes

*Note*: Denominators vary due to missing information. Numbers do not include F2‐II‐2/F7‐II‐2 as molecular confirmation is still awaited in those individuals. A more detailed breakdown is available in Table S3.

Sajan et al. described overlapping dysmorphic features for both individuals reported in 2018.^5^ In the cohort described here, consented images are available for six individuals and we show that subtle dysmorphic features can include flat face, high and broad forehead and hypertelorism. Several other variable features also overlap with those seen in Noonan syndrome (NS) and include pectus excavatum and low‐set ears. Indeed, Face2Gene analysis proposes NS as the most likely diagnosis for 50% of cases (Table S3). Differential diagnoses for this condition may also include Robinow syndrome, which was proposed as a clinical diagnosis in F1/F6/F7. Proptosis, depressed nasal bridge, thick arched eyebrows, a long philtrum and thin upper lips were seen in the younger patients but absent in the adult patient, which could suggest the evolution of the facial features with age (Figure 2F–K). Hearing loss was reported for both sisters in Family 3 and for F5‐II‐10. This feature was also seen in one of the cases reported previously^5^ suggesting this to be a recurring characteristic of this condition. Downslanting palpebral fissures, brachial cleft defect and hoarse voice, were seen in F5‐II‐10 and was previously reported in one of the patients of Sajan et al. Sloping shoulders were seen in three of the patients reported here. Short thumbs and clinodactyly or shortening of the third/fifth digit were seen in multiple patients. In the lower limbs, patellofemoral joint dislocation was observed in three cases and genu varum was seen in F5‐II‐10. Notably, this individual is the first adult case to be described and a more detailed clinical summary is provided in Data S1. Following radiological assessment, short humeri were noted in the skeletal surveys available for 6 of 8 cases and radiological data was available for review in five families (Figure 2L–T).

One limitation of this study is that for p.(Leu262Arg), further evidence supporting pathogenicity would be desirable. Although in silico analysis predicted that the variant may impact protein stability, this should ideally be confirmed experimentally. Segregation studies on F5‐II‐10's 9 siblings (should DNA become available) may also be used to help support pathogenicity. The same variant in Family 6 was originally assessed as a VUS (ClinVar, SCV002127124.1). However, based on clinical judgement and the finding of the same variant in F5, the patient is now being treated as if they have a molecular diagnosis.

As four of the families were recruited from large clinical databases, we were able to estimate the likely prevalence of this condition in suitable patient populations. There was a 2 of 253 incidence of *PKDCC* positive cases amongst patients recruited to the 100 kGP (v15, 26 May 2022) due to unexplained skeletal dysplasia. Families 3 and 4 were identified from the GeneDx variant database (v14 April 2021) containing exome data for 1442 individuals with skeletal dysplasia phenotypes. In combination (4/1695), and given that skeletal dysplasias themselves are estimated to have a prevalence of 3.2 per 10 000 births,^9^ our results are consistent with this condition being extremely rare in humans.

Three of the variants identified lie in a low complexity GC‐rich region (p.Gly74‐Gly131). In Family 2, this contributed to p.(Pro77Alafs*95) being called as a low‐confidence variant (‘SC;badReads’), explaining why it had initially been missed. Genomic regions with low complexity can be highly mutagenic and are often hard to analyse. We speculate that patients with upper‐limb rhizomelia in whom a single heterozygous pathogenic allele in *PKDCC* has been uncovered may benefit from closer scrutiny of this region.

In summary, we identified eight affected individuals from seven independent families with biallelic loss of function variants in *PKDCC*. These data help delineate the phenotypic spectrum associated with this ultra‐rare condition and will allow clinical laboratories to have more confidence in interpreting variants in this gene.

## AUTHOR CONTRIBUTIONS

Alistair T. Pagnamenta and Jenny C. Taylor conceived the project. Genomics England Research Consortium, Benito Banos‐Pinero, Karolina Pawliczak, and Reza Maroofian generated genetic data. Alistair T. Pagnamenta, Ingrid M. Wentzensen, Maria J. Guillen Sacoto, Reza Maroofian, Tracy Lester, Fan Xia, and Hossein Najmabadi performed data analysis. Matteo Ferla performed structural‐modelling. Francis Jeshira Reynoso Santos, Alesky Caffo, Rebecca S. Belles, Bonnie Anne Salbert, Ana Lucia Yanez‐Felix, Camilo E. Villarroel‐Cortes, Khowla Al Fayez, Amal Al Hashem, Deborah Shears, Melita Irving, and Ariana Kariminejad recruited families/collected clinical data. Amaka C. Offiah reviewed radiographs. Alistair T. Pagnamenta, Rebecca S. Belles, and Ariana Kariminejad drafted the manuscript.

## FUNDING INFORMATION

Supported by the MRC (MR/W01761X/1), the Wellcome Trust (203141/Z/16/Z) and the Oxford NIHR Biomedical Research Centre.

## CONFLICT OF INTEREST STATEMENT

Ingrid M. Wentzensen and Maria J. Guillen Sacoto are employees of GeneDx, LLC.

### PEER REVIEW

The peer review history for this article is available at https://www.webofscience.com/api/gateway/wos/peer-review/10.1111/cge.14324.

## Supporting information


**Data S1:** Supporting Information

**Table S3:** Variant information and clinical details for individuals with biallelic variants in PKDCC.

## Data Availability

100 kGP data are in the National Genomic Research Library (https://doi.org/10.6084/m9.figshare.4530893.v6).
