# Supplementary material for: The prevalence and phenotypic range associated with biallelic PKDCC variants
Source: Clin Genet. 2023 Mar 10;104(1):121–6. doi: 10.1111/cge.14324 (PMC10952701; doi:10.1111/cge.14324)
Supplement: Supplementary file 1 — Data S1: Supporting Information [file CGE-104-121-s002.docx]

**The prevalence and phenotypic range associated with biallelic *PKDCC* variants *Supplementary Material***

**The Genomics England Research Consortium†**

John C. Ambrose^1^; Prabhu Arumugam^1^; Roel Bevers^1^; Marta Bleda^1^; Freya Boardman-Pretty^1,2^; Christopher R. Boustred^1^; Helen Brittain^1^; Mark J. Caulfield^1,2^; Georgia C. Chan^1^; Greg Elgar^1,2^; Tom Fowler^1^; Adam Giess^1^; Angela Hamblin^1^; Shirley Henderson^1,2^; Tim J. P. Hubbard^1^; Rob Jackson^1^; Louise J. Jones^1,2^; Dalia Kasperaviciute^1,2^; Melis Kayikci^1^; Athanasios Kousathanas^1^; Lea Lahnstein^1^; Sarah E. A. Leigh^1^; Ivonne U. S. Leong^1^; Javier F. Lopez^1^; Fiona Maleady-Crowe^1^; Meriel McEntagart^1^; Federico Minneci^1^; Loukas Moutsianas^1,2^; Michael Mueller^1,2^; Nirupa Murugaesu^1^; Anna C. Need^1,2^; Peter O’Donovan^1^; Chris A. Odhams^1^; Christine Patch^1,2^; Mariana Buongermino Pereira^1^; Daniel Perez-Gil^1^; John Pullinger^1^; Tahrima Rahim^1^; Augusto Rendon^1^; Tim Rogers^1^; Kevin Savage^1^; Kushmita Sawant^1^; Richard H. Scott^1^; Afshan Siddiq^1^; Alexander Sieghart^1^; Samuel C. Smith^1^; Alona Sosinsky^1,2^; Alexander Stuckey^1^; Mélanie Tanguy^1^; Ana Lisa Taylor Tavares^1^; Ellen R. A. Thomas^1,2^; Simon R. Thompson^1^; Arianna Tucci^1,2^; Matthew J. Welland^1^; Eleanor Williams^1^; Katarzyna Witkowska^1,2^; Suzanne M. Wood^1,2^.

1. Genomics England, London, UK

2. William Harvey Research Institute, Queen Mary University of London, London, EC1M 6BQ, UK.

†The 100kGP is funded by the NIHR and NHS England. The Wellcome Trust, Cancer Research UK and the MRC also funded research infrastructure**.**

**Structural modelling methods**

The difference in Gibbs free energy at the protein level was predicted using Venus (Ferla et al., 2022) in combination with an AlphaFold2 model (Jumper et al., 2021). Further modelling and scoring was done with ColabFold (Mirdita et al., 2022), PyRosetta (Chaudhury et al., 2010) and a ligand-bound homologue structure (PDB:1GY3) (Cook et al., 2002). The interactive model was made in MichelaNGLo (Ferla et al., 2020).

**Methodological details and clinical summary for first adult case of *PKDCC-*associated skeletal dysplasia to be described**

In Family 5, fragmented blood DNA was enriched for the coding regions of all known human genes. Additional custom designed probes targeted established pathogenic variants in noncoding regions as well as mtDNA. The Illumina NovaSeq was used for generating paired-end 150bp sequencing reads. Alignment to GRCh37 used BWA and variants were called with GATK. Variant annotation and filtering used allele frequencies from gnomAD (v2.1.1), the Iranome catalogue of genomic variation (www.iranome.ir) and the 1000 Genomes Project.

Individual F5-II-10 is an Iranian male in his 40s and one of 10 offspring. His parents are related and parents and all 9 siblings are healthy. Information regarding birth is not available, but short stature and mild dysmorphic features were noted from infancy. Milestones were somewhat delayed, walked and started saying first words at the age of 2 years. He had a brachial cleft defect (fistula) which was repaired in childhood. His measurements are as follows: height weight and head circumference 157cm, 77kg and 57cm respectively. At examination he has mild facial dysmorphism including high and broad forehead, hypertelorism, downslanting palpebral fissures, flat face, sloping shoulders, rhizomelic shortening of upper and lower limbs, genu varum, patellofemoral dislocation, incomplete supination in both arms, short thumbs, clinodactyly of left fifth finger and only one interphalangeal crease on both 5th fingers (Figure S1) and a protuberant abdomen. He complained of chronic joint pain. X-rays and clinical pictures were sent to International Skeletal Dysplasia Registry and bi-temporal narrowing with normal sutures were noted in the skull. The spine showed slightly high vertebral bodies, mild lumbosacral interpeculate narrowing, and degeneration of thoracic spine. The epiphysial and diaphyseal regions were normal in spite of rhizomelic appearance of limbs. Mild Patterson-Lowry dysplasia, omodysplasia were suggested as differential diagnoses. Mild metaphyseal dysplasias could not be excluded.

**Supplementary discussion – ROH region analysis**

Pathogenic variants are overrepresented in the largest ROHs and it has been proposed that lying in one of the top 10 such regions can be used as evidence supporting pathogenicity (Wakeling et al., 2019). Whilst in Family 1 the *PDKCC* frameshift lay within the 2^nd^ largest ROH, in Family 2 the frameshift was within a much smaller ROH region, ranked only 24^th^ (Table S2). These results highlight that in some cases it can be worthwhile extending assessment of ROHs to include those <1Mb in size.

**Table S1:** Details of 70 genes (including *PKDCC*) listed as green in the current Skeletal Dysplasia panel from PanelApp (v2.207), but which were not coded as green in the panel applied at time when the initial clinical tiering was performed on Family 2 (v1.144). Table compiled using the multi-symbol checker tool in [www.genenames.org](http://www.genenames.org). †listed in PanelApp under previous symbol, *FAM46A.*

| **Approved symbol** | **Approved name** | **HGNC ID** | **Location** |
| --- | --- | --- | --- |
| *ADAMTS10* | ADAM metallopeptidase with thrombospondin type 1 motif 10 | HGNC:13201 | 19p13.2 |
| *ADAMTS17* | ADAM metallopeptidase with thrombospondin type 1 motif 17 | HGNC:17109 | 15q26.3 |
| *ALX1* | ALX homeobox 1 | HGNC:1494 | 12q21.31 |
| *ARCN1* | archain 1 | HGNC:649 | 11q23.3 |
| *ARL6* | ADP ribosylation factor like GTPase 6 | HGNC:13210 | 3q11.2 |
| *B3GLCT* | beta 3-glucosyltransferase | HGNC:20207 | 13q12.3 |
| *BBS1* | Bardet-Biedl syndrome 1 | HGNC:966 | 11q13.2 |
| *BBS10* | Bardet-Biedl syndrome 10 | HGNC:26291 | 12q21.2 |
| *BBS12* | Bardet-Biedl syndrome 12 | HGNC:26648 | 4q27 |
| *BBS2* | Bardet-Biedl syndrome 2 | HGNC:967 | 16q13 |
| *BBS4* | Bardet-Biedl syndrome 4 | HGNC:969 | 15q24.1 |
| *BBS5* | Bardet-Biedl syndrome 5 | HGNC:970 | 2q31.1 |
| *BBS7* | Bardet-Biedl syndrome 7 | HGNC:18758 | 4q27 |
| *BBS9* | Bardet-Biedl syndrome 9 | HGNC:30000 | 7p14.3 |
| *COG1* | component of oligomeric golgi complex 1 | HGNC:6545 | 17q25.1 |
| *COG4* | component of oligomeric golgi complex 4 | HGNC:18620 | 16q22.1 |
| *COPB2* | COPI coat complex subunit beta 2 | HGNC:2232 | 3q23 |
| *CREB3L1* | cAMP responsive element binding protein 3 like 1 | HGNC:18856 | 11p11.2 |
| *CSGALNACT1* | chondroitin sulfate N-acetylgalactosaminyltransferase 1 | HGNC:24290 | 8p21.3 |
| *CYP2R1* | cytochrome P450 family 2 subfamily R member 1 | HGNC:20580 | 11p15.2 |
| *DHCR7* | 7-dehydrocholesterol reductase | HGNC:2860 | 11q13.4 |
| *DPAGT1* | dolichyl-phosphate N-acetylglucosaminephosphotransferase 1 | HGNC:2995 | 11q23.3 |
| *FN1* | fibronectin 1 | HGNC:3778 | 2q35 |
| *FZD2* | frizzled class receptor 2 | HGNC:4040 | 17q21.31 |
| *HS2ST1* | heparan sulfate 2-O-sulfotransferase 1 | HGNC:5193 | 1p22.3 |
| *IFIH1* | interferon induced with helicase C domain 1 | HGNC:18873 | 2q24.2 |
| *KAT6B* | lysine acetyltransferase 6B | HGNC:17582 | 10q22.2 |
| *KDELR2* | KDEL endoplasmic reticulum protein retention receptor 2 | HGNC:6305 | 7p22.1 |
| *KIAA0753* | KIAA0753 | HGNC:29110 | 17p13.1 |
| *KMT2D* | lysine methyltransferase 2D | HGNC:7133 | 12q13.12 |
| *LRRK1* | leucine rich repeat kinase 1 | HGNC:18608 | 15q26.3 |
| *LTBP1* | latent transforming growth factor beta binding protein 1 | HGNC:6714 | 2p22.3 |
| *MASP1* | MBL associated serine protease 1 | HGNC:6901 | 3q27.3 |
| *MBTPS1* | membrane bound transcription factor peptidase, site 1 | HGNC:15456 | 16q23.3-q24.1 |
| *MESD* | mesoderm development LRP chaperone | HGNC:13520 | 15q25.1 |
| *MKKS* | MKKS centrosomal shuttling protein | HGNC:7108 | 20p12.2 |
| *MTX2* | metaxin 2 | HGNC:7506 | 2q31.1 |
| *MYO18B* | myosin XVIIIB | HGNC:18150 | 22q12.1 |
| *NBAS* | NBAS subunit of NRZ tethering complex | HGNC:15625 | 2p24.3 |
| *NPR3* | natriuretic peptide receptor 3 | HGNC:7945 | 5p13.3 |
| *NXN* | nucleoredoxin | HGNC:18008 | 17p13.3 |
| *P4HB* | prolyl 4-hydroxylase subunit beta | HGNC:8548 | 17q25.3 |
| *PAX3* | paired box 3 | HGNC:8617 | 2q36.1 |
| *PIK3C2A* | phosphatidylinositol-4-phosphate 3-kinase catalytic subunit type 2 alpha | HGNC:8971 | 11p15.1 |
| *PISD* | phosphatidylserine decarboxylase | HGNC:8999 | 22q12.2 |
| *PKDCC* | protein kinase domain containing, cytoplasmic | HGNC:25123 | 2p21 |
| *POLR1B* | RNA polymerase I subunit B | HGNC:20454 | 2q14.1 |
| *PRKG2* | protein kinase cGMP-dependent 2 | HGNC:9416 | 4q21.21 |
| *RAB33B* | RAB33B, member RAS oncogene family | HGNC:16075 | 4q31.1 |
| *RINT1* | RAD50 interactor 1 | HGNC:21876 | 7q22.3 |
| *RPL13* | ribosomal protein L13 | HGNC:10303 | 16q24.3 |
| *SCUBE3* | signal peptide, CUB domain and EGF like domain containing 3 | HGNC:13655 | 6p21.3 |
| *SGMS2* | sphingomyelin synthase 2 | HGNC:28395 | 4q25 |
| *SLC10A7* | solute carrier family 10 member 7 | HGNC:23088 | 4q31.22 |
| *SLC34A1* | solute carrier family 34 member 1 | HGNC:11019 | 5q35.3 |
| *SLC35C1* | solute carrier family 35 member C1 | HGNC:20197 | 11p11.2 |
| *SMAD6* | SMAD family member 6 | HGNC:6772 | 15q22.31 |
| *SP7* | Sp7 transcription factor | HGNC:17321 | 12q13.13 |
| *SPARC* | secreted protein acidic and cysteine rich | HGNC:11219 | 5q33.1 |
| *TAPT1* | transmembrane anterior posterior transformation 1 | HGNC:26887 | 4p15.32 |
| *TENT5A†* | terminal nucleotidyltransferase 5A | HGNC:18345 | 6q14.1 |
| *TONSL* | tonsoku like, DNA repair protein | HGNC:7801 | 8q24.3 |
| *TTC8* | tetratricopeptide repeat domain 8 | HGNC:20087 | 14q31.3 |
| *UFSP2* | UFM1 specific peptidase 2 | HGNC:25640 | 4q35.1 |
| *UNC45A* | unc-45 myosin chaperone A | HGNC:30594 | 15q26.1 |
| *VDR* | vitamin D receptor | HGNC:12679 | 12q13.11 |
| *WBP11* | WW domain binding protein 11 | HGNC:16461 | 12p12.3 |
| *WDPCP* | WD repeat containing planar cell polarity effector | HGNC:28027 | 2p15 |
| *ZNF687* | zinc finger protein 687 | HGNC:29277 | 1q21.3 |
| *ZSWIM6* | zinc finger SWIM-type containing 6 | HGNC:29316 | 5q12.1 |

**Table S2:** The top 30 regions of homozygosity (ROH) detected in F2-II-1. Genomic coordinates are based on GRCh38. *PKDCC* lies in the 24^th^ largest region of ROH.

| **Chr** | **Start** | **End** | **Size** | **Notes** |
| --- | --- | --- | --- | --- |
| chr19 | 23,488,180 | 28,066,002 | 4,577,822 | spans centromere |
| chr5 | 46,300,825 | 50,866,899 | 4,566,074 | spans centromere |
| chr6 | 156,142,805 | 160,046,857 | 3,904,052 |  |
| chr6 | 100,251,398 | 103,991,552 | 3,740,154 |  |
| chr2 | 22,980,263 | 25,499,514 | 2,519,251 |  |
| chr6 | 31,869,050 | 34,217,234 | 2,348,184 |  |
| chr4 | 133,611,420 | 135,759,592 | 2,148,172 |  |
| chr13 | 25,890,244 | 28,015,357 | 2,125,113 |  |
| chr8 | 72,328,687 | 74,450,087 | 2,121,400 |  |
| chr12 | 104,453,231 | 106,307,179 | 1,853,948 |  |
| chr4 | 180,046,650 | 181,701,564 | 1,654,914 |  |
| chr13 | 107,867,650 | 109,489,846 | 1,622,196 |  |
| chr6 | 60,971,241 | 62,264,102 | 1,292,861 |  |
| chr10 | 14,981,401 | 16,025,241 | 1,043,840 |  |
| chr6 | 29,387,425 | 30,397,962 | 1,010,537 |  |
| chr10 | 13,048,714 | 13,965,577 | 916,863 |  |
| chr6 | 77,191,417 | 78,083,536 | 892,119 |  |
| chr7 | 63,866,668 | 64,752,443 | 885,775 |  |
| chr11 | 45,227,763 | 46,090,024 | 862,261 |  |
| chr1 | 214,107,189 | 214,958,747 | 851,558 |  |
| chr8 | 138,242,331 | 139,080,380 | 838,049 |  |
| chr4 | 127,537,013 | 128,359,060 | 822,047 |  |
| chr11 | 88,599,364 | 89,403,556 | 804,192 |  |
| chr2 | 41,291,367 | 42,082,273 | 790,906 | This region contains *PKDCC* |
| chr2 | 195,416,490 | 196,204,369 | 787,879 |  |
| chr13 | 55,064,375 | 55,844,725 | 780,350 |  |
| chr15 | 88,632,195 | 89,405,978 | 773,783 |  |
| chr15 | 83,808,953 | 84,551,434 | 742,481 |  |
| chr15 | 76,181,349 | 76,914,935 | 733,586 |  |
| chr18 | 14,357,380 | 15,046,605 | 689,225 |  |


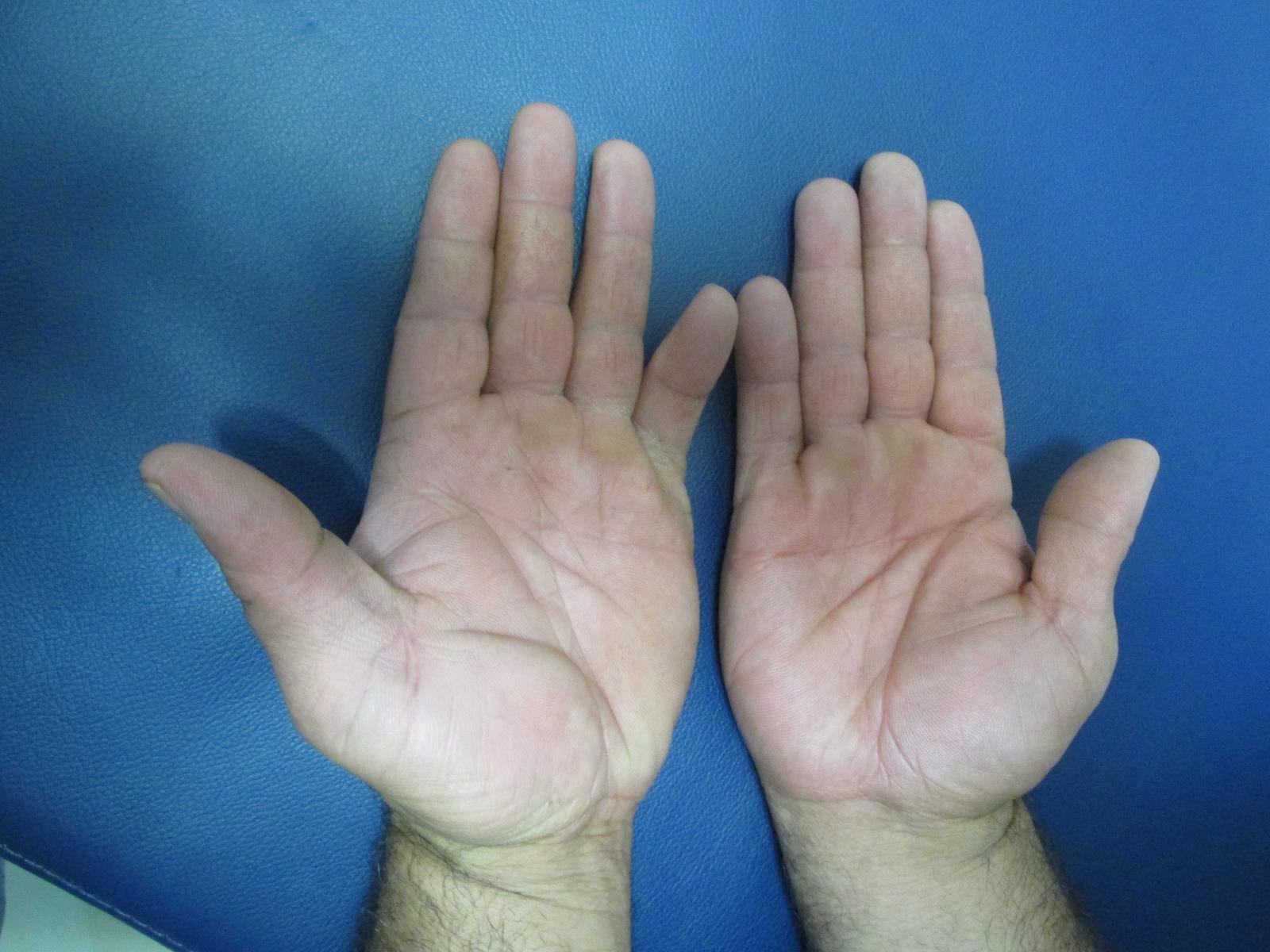


**Figure S1:** Photographs of individual F5-II-10 showing short thumbs, clinodactyly of fifth fingers and single digital crease of fifth fingers.


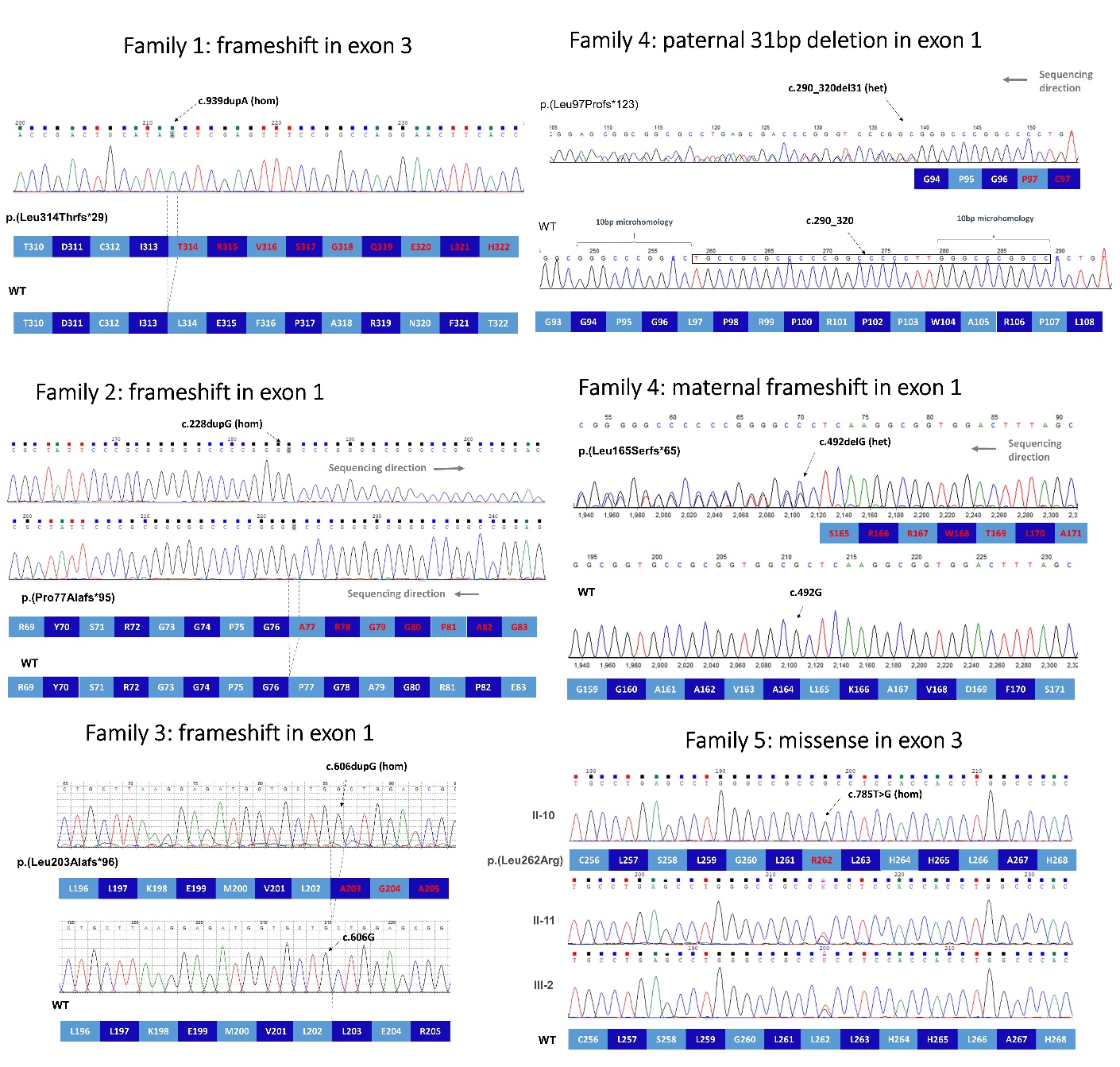


**Figure S2:** Sanger validation data for variants described in Families 1-5 in this study. Labelling is using gene coordinates based on NM_138370.3. For Family 6, we note that Invitae typically only validate variants by orthogonal approaches where it is required (Lincoln et al., 2019) but in this case it was done using the PacBio platform. For Family 7, PCR-Sanger validation was performed but data is not available to review.


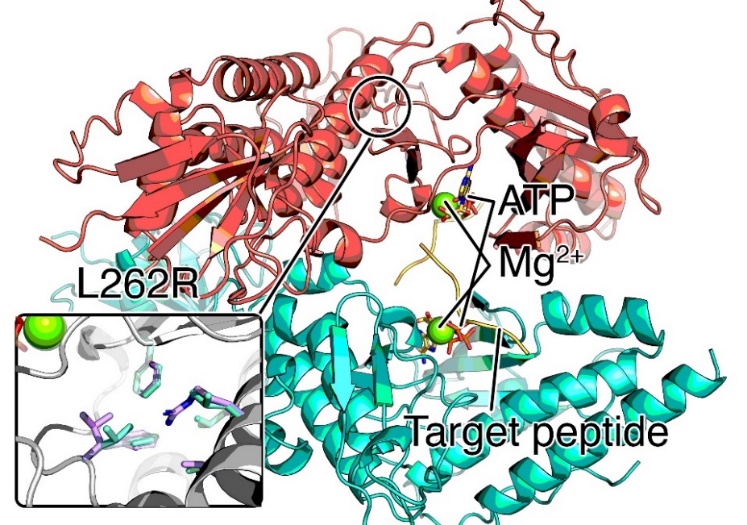


**Figure S3:** The model shows a PKDCC dimer with ATP, magnesium ion and target peptide as a proxy for the larger target protein. L262 is a buried residue 14Å away from the ATP/magnesium cofactors and packed with residues 279, 284 and 293, which are on structural elements involved in binding these cofactors.

References

Chaudhury, S., Lyskov, S., & Gray, J. J. (2010). PyRosetta: a script-based interface for implementing molecular modeling algorithms using Rosetta. *Bioinformatics*, *26*(5), 689-691. <https://doi.org/10.1093/bioinformatics/btq007>

Cook, A., Lowe, E. D., Chrysina, E. D., Skamnaki, V. T., Oikonomakos, N. G., & Johnson, L. N. (2002). Structural studies on phospho-CDK2/cyclin A bound to nitrate, a transition state analogue: implications for the protein kinase mechanism. *Biochemistry*, *41*(23), 7301-7311. <https://doi.org/10.1021/bi0201724>

Ferla, M. P., Pagnamenta, A. T., Damerell, D., Taylor, J. C., & Marsden, B. D. (2020). MichelaNglo: sculpting protein views on web pages without coding. *Bioinformatics*, *36*(10), 3268-3270. <https://doi.org/10.1093/bioinformatics/btaa104>

Ferla, M. P., Pagnamenta, A. T., Koukouflis, L., Taylor, J. C., & Marsden, B. D. (2022). Venus: Elucidating the Impact of Amino Acid Variants on Protein Function Beyond Structure Destabilisation. *J Mol Biol*, *434*(11), 167567. <https://doi.org/10.1016/j.jmb.2022.167567>

Jumper, J., Evans, R., Pritzel, A., Green, T., Figurnov, M., Ronneberger, O., Tunyasuvunakool, K., Bates, R., Zidek, A., Potapenko, A., Bridgland, A., Meyer, C., Kohl, S. A. A., Ballard, A. J., Cowie, A., Romera-Paredes, B., Nikolov, S., Jain, R., Adler, J., . . . Hassabis, D. (2021). Highly accurate protein structure prediction with AlphaFold. *Nature*, *596*(7873), 583-589. <https://doi.org/10.1038/s41586-021-03819-2>

Lincoln, S. E., Truty, R., Lin, C. F., Zook, J. M., Paul, J., Ramey, V. H., Salit, M., Rehm, H. L., Nussbaum, R. L., & Lebo, M. S. (2019). A Rigorous Interlaboratory Examination of the Need to Confirm Next-Generation Sequencing-Detected Variants with an Orthogonal Method in Clinical Genetic Testing. *J Mol Diagn*, *21*(2), 318-329. <https://doi.org/10.1016/j.jmoldx.2018.10.009>

Mirdita, M., Schutze, K., Moriwaki, Y., Heo, L., Ovchinnikov, S., & Steinegger, M. (2022). ColabFold: making protein folding accessible to all. *Nat Methods*, *19*(6), 679-682. <https://doi.org/10.1038/s41592-022-01488-1>

Wakeling, M. N., Laver, T. W., Wright, C. F., De Franco, E., Stals, K. L., Patch, A. M., Hattersley, A. T., Flanagan, S. E., Ellard, S., & Study, D. D. D. (2019). Homozygosity mapping provides supporting evidence of pathogenicity in recessive Mendelian disease. *Genet Med*, *21*(4), 982-986. <https://doi.org/10.1038/s41436-018-0281-4>
